# Innovating Equity-Focused Strategies for Hypertensive Disorders of Pregnancy

**DOI:** 10.1016/j.jacadv.2025.102429

**Published:** 2025-12-18

**Authors:** Stephanie Porter-Plummer

**Affiliations:** Undergraduate Nursing, Florida International University, Miami, Florida, USA

**Keywords:** gestational hypertension, high blood pressure, hypertension, hypertensive disorder in pregnancy, preeclampsia

Hypertensive disorders of pregnancy complicate up to 15% of pregnancies in the United States and remain among the leading causes of maternal morbidity and mortality. The burden is not equally shared: Black women face a threefold higher risk of maternal death compared to White women, underscoring the intersection of clinical and equity challenges. In their state-of-the-art review, Radparvar et al^1^ highlight both the limitations of current practice and the promise of innovative strategies to align care with risk.

One of the most pressing issues in hypertensive disease in pregnancy management is the timing of surveillance. Acute hypertensive crises and seizures often occur within 3 to 6 days postpartum, yet routine evaluation is scheduled at 6 weeks. This mismatch contributes to preventable readmissions and deaths, nearly 60% of which are considered avoidable. Reimagining postpartum care requires early follow-up within the first week after discharge, patient education and warning signs, and structured systems for timely blood pressure evaluation and treatment.

Blood pressure self-monitoring improves detection of acute hypertension when paired with automated data transfer and provider engagement. Trials such as SNAP-HT (Self-Management of Postnatal Hypertension trial) demonstrate reduced blood pressure and decreased treatment burden when self-monitoring is integrated into structured care models.

Telemedicine expands postpartum surveillance, particularly for patients facing barriers to in-person care. Studies demonstrate telehealth visit attendance rates more than double those of clinic-based care, with text-based blood pressure monitoring eliminating racial disparities in follow-up adherence in some cohorts.

Community health workers extend culturally concordant, trust-based support into communities, increasing prenatal and postpartum engagement. Their integration into cardio-obstetrics teams can help bridge health systems with historically underserved populations.

Together, these strategies provide a framework for patient-centered, scalable care that better aligns with the risk trajectory of hypertensive disorders in pregnancy.

While innovation in surveillance is critical, delivery mode remains a pivotal decision point. Vaginal delivery is generally favored due to lower surgical morbidity. However, for patients with severe preeclampsia or eclampsia, cesarean delivery may represent the safer route. The physiologic stress of labor pain, contractions, and Valsalva maneuvers can precipitate hypertensive crises or seizures. Cesarean delivery, a controlled environment with anesthesia, antihypertensive therapy, and magnesium prophylaxis readily available, may mitigate these risks. Recent evidence underscores the nuance required. Watkins et al^2^ found that cesarean delivery should not be universally applied to patients with preeclampsia, with outcomes supporting individualized decision-making. Conversely, Bushman et al^3^ reported that induction of labor was associated with lower maternal morbidity than pre labor cesarean section in selected cases. Observational studies by Pasokpuckdee et al^4^ demonstrate that women with higher blood pressures are more likely to undergo cesarean delivery, and population-level data confirm higher cesarean delivery rates among preeclamptic women. These findings suggest that, while cesarean delivery should not replace standard obstetric indications, it may serve as a protective strategy against hypertensive emergencies in select high-risk patients.

Addressing hypertensive disorders in pregnancy requires more than clinical innovation. Systemic inequities remain central drivers of disparate outcomes. Black and Latina women not only face higher risk of preeclampsia but also encounter barriers such as transportation challenges, insurance gaps, and implicit bias in care delivery. Telehealth and community health workers integration may mitigate these disparities by extending access, offering culturally tailored education, and ensuring timely postpartum surveillance. Yet, digital divides in rural areas and lack of sustainable financing for community health worker programs highlights the need for supportive policy and infrastructure investment.

Radparvar et al^1^ advance the conversation by reframing hypertensive disorders in pregnancy as both a clinical and equity crisis. Integrating self-monitoring, telemedicine, and community health workers provides a road map for aligning care with risk and reducing preventable deaths. At the same time, delivery planning must be individualized, with cesarean delivery reserved as a protective strategy for those at highest risk of hypertensive crisis or seizures. Future research should prioritize diverse cohorts, cost-effectiveness analysis, and implementation frameworks that institutionalize equity as a core principle. With nearly 60% of hypertensive-disorders-related maternal deaths considered preventable, the opportunity and obligation for transformation is urgent.

## Funding support and author disclosures

The author has reported that they have no relationships relevant to the contents of this paper to disclose.

## References

[1] Radparvar A.A., Vani K., Fiori K. (2024). Hypertensive disorders of pregnancy: innovative management strategies. JACC Adv.

[2] Watkins V.Y., Federspiel J.J., Sugrue R.P. (2024). Maternal outcomes by mode of delivery among pregnant patients with eclampsia: a retrospective cohort study. Am J Obstet Gynecol MFM.

[3] Bushman E.T., Brar A., Contag S., Dudley D.J., Zera C.A. (2023). Outcomes of induction vs prelabor cesarean delivery. Am J Obstet Gynecol MFM.

[4] Pasokpuckdee K., Boriboonhirunsarn D. (2023). Incidence of preeclampsia and cesarean section rate according to the robson classification. Cureus.

